# Quantification of abnormal QRS peaks predicts response to cardiac resynchronization therapy and tracks structural remodeling

**DOI:** 10.1371/journal.pone.0217875

**Published:** 2019-06-06

**Authors:** Adrian M. Suszko, Sachin Nayyar, Andreu Porta-Sanchez, Moloy Das, Arnold Pinter, Eugene Crystal, George Tomlinson, Rupin Dalvi, Vijay S. Chauhan

**Affiliations:** 1 Peter Munk Cardiac Centre, University Health Network, Toronto, ON, Canada; 2 Department of Cardiology, Freeman Hospital, Newcastle upon Tyne, United Kingdom; 3 Division of Cardiology, St. Michael’s Hospital, Toronto, ON, Canada; 4 Cardiology Division, Sunnybrook Health Sciences Centre, Toronto, ON, Canada; University of Minnesota, UNITED STATES

## Abstract

**Background:**

Although QRS duration (QRSd) is an important determinant of cardiac resynchronization therapy (CRT) response, non-responder rates remain high. QRS fragmentation can also reflect electrical dyssynchrony. We hypothesized that quantification of abnormal QRS peaks (QRSp) would predict CRT response.

**Methods:**

Forty-seven CRT patients (left ventricular ejection fraction = 23±7%) were prospectively studied. Digital 12-lead ECGs were recorded during native rhythm at baseline and 6 months post-CRT. For each precordial lead, QRSp was defined as the total number of peaks detected on the unfiltered QRS minus those detected on a smoothed moving average template QRS. CRT response was defined as >5% increase in left ventricular ejection fraction post-CRT.

**Results:**

Sixty-percent of patients responded to CRT. Baseline QRSd was similar in CRT responders and non-responders, and did not change post-CRT regardless of response. Baseline QRSp was greater in responders than non-responders (9.1±3.5 vs. 5.9±2.2, p = 0.001) and decreased in responders (9.2±3.6 vs. 7.9±2.8, p = 0.03) but increased in non-responders (5.5±2.3 vs. 7.5±2.8, p = 0.049) post-CRT. In multivariable analysis, QRSp was the only independent predictor of CRT response (Odds Ratio [95% Confidence Interval]: 1.5 [1.1–2.1], p = 0.01). ROC analysis revealed QRSp (area under curve = 0.80) to better discriminate response than QRSd (area under curve = 0.67). Compared to QRSd ≥150ms, QRSp ≥7 identified response with similar sensitivity but greater specificity (74 vs. 32%, p<0.05). Amongst patients with QRSd <150ms, more patients with QRSp ≥7 responded than those with QRSp <7 (75 vs. 0%, p<0.05).

**Conclusions:**

Our novel automated QRSp metric independently predicts CRT response and decreases in responders. Electrical dyssynchrony assessed by QRSp may improve CRT selection and track structural remodeling, especially in those with QRSd <150ms.

## Introduction

Cardiac resynchronization therapy (CRT) restores electromechanical left ventricular (LV) synchrony and has been shown to reverse structural remodeling and improve clinical outcomes in heart failure patients with New York Heart Association (NYHA) class II-III function, LV ejection fraction (LVEF) <35%, and QRS duration (QRSd) >120ms [1]. Yet, a large proportion of these patients (~30–40%) do not respond to CRT, often due to the presence of minimal electromechanical dyssynchrony or suboptimal LV lead pacing/placement [2]. In view of this, targeted LV lead implantation to sites of latest electrical or mechanical activation has improved CRT response rate [3]. However, the assessment of mechanical activation time and dyssynchrony based on echocardiographic-derived measures of regional strain and wall motion can be limited by large observer variability, which may account for the lack of consistent improvement in CRT response when using these metrics for patient selection. In contrast, the evaluation of electrical dyssynchrony using QRSd and bundle branch block (BBB) morphology appears more reliable and the CRT response rate increases in patients with more prolonged QRSd and left BBB (LBBB) [4]. Nonetheless, LV activation timing can still be quite heterogeneous for any given QRSd or BBB morphology due to varying spatial/transmural scar mass, scar border zones of slow conduction and lines of functional conduction block [5]. Structural remodeling in this manner can change the direction of activating wavefronts in addition to delaying LV activation time, which can manifest on the surface 12-lead ECG as QRS fragmentation [6].

The presence of QRS fragmentation has been shown to predict mortality and sudden cardiac death in patients with coronary artery disease and cardiomyopathy [7]. QRS fragmentation is also associated with echocardiographically-derived ventricular dyssynchrony [8,9], but its ability to predict CRT response has been inconsistent [10,11]. A potential limitation of these CRT studies is the qualitative (i.e. present or absent) evaluation of QRS fragmentation (fQRS) based on manually-defined large intra-QRS deflection from a low resolution standard 12-lead ECG, which may not discern more localized, yet dyssynchronous myopathic regions. In view of this, we have developed a fully-automated, validated algorithm to quantify small QRS deflections from higher resolution extended 12-lead ECG recordings [12]. The aggregate number of these abnormal QRS peaks (QRSp) do not correlate with QRSd and independently predict ventricular tachyarrhythmias in cardiomyopathy patients eligible for primary prevention implantable cardioverter defibrillator (ICD) [13]. In the present study, we hypothesized that quantification of QRSp will be more predictive of functional response to CRT than QRSd, BBB morphology or fQRS. Our objective was to prospectively evaluate the utility of QRSp in identifying functional CRT responders and tracking structural remodeling after CRT.

## Methods

### Study population and CRT implant

Forty-seven consecutive patients with ischemic or non-ischemic dilated cardiomyopathy undergoing CRT-defibrillator implantation (either *de novo* or upgrade from single or dual chamber ICD), according to current heart failure management guidelines were prospectively enrolled [1]. Patients in complete heart block were excluded. Prior to consideration of CRT, all patients had an LVEF ≤35%, native QRSd ≥120ms, and had received optimal medical therapy for at least 3 months. All patients were required to have conventional LBBB regardless of QRSd provided it was ≥120ms, or non-LBBB with QRSd ≥150ms.

According to standard implantation procedures, all patients had a high voltage right ventricular (RV) lead placed in the RV apex and an LV lead placed in a postero-lateral or lateral tributary of the coronary sinus. Septal and apical LV stimulation were avoided where feasible. All but three patients with persistent atrial fibrillation (AF) underwent transvenous implantation of an atrial lead. Permanent CRT programming of AV delay, LV to RV delay and the LV stimulation vector was optimized for each patient as per the discretion of the treating electrophysiologist based on a combination of factors that included intrinsic PR interval, paced QRS morphology and QRSd after CRT. The study was approved by the local Research Ethics Boards and informed written consent was obtained from all subjects.

### QRSd, fQRS and QRSp assessment

A baseline ECG in native QRS rhythm was collected within the first 3 months of CRT for all patients and a follow-up study of native QRS rhythm was performed after 6 months of CRT in 38 patients. At each study, the skin was carefully prepared before recording high-resolution 12-lead ECGs for 3 minutes using a digital 12-lead Holter monitor (CardioMem CM 3000-12BT, Getemed Inc., Teltow, Germany) at 1024Hz sampling rate (0.05-120Hz analogue bandwidth, ±6 mV voltage range, 12-bit digital resolution, 2.9μV least significant bit). To minimize ECG noise, patients were required to lie still in the supine position with their hands at their sides for the duration of the recording.

Intrinsic QRSd was measured as the difference between the earliest QRS onset and the latest QRS offset on the baseline digital 12-lead ECG. The presence of intrinsic fQRS was assessed according to published criteria from a paper print out of the digital ECG using standard 12-lead ECG technical specifications (25mm/s, 10 mm/mV, 0.01–150 Hz bandpass filter) [14]. Intrinsic QRSp was automatically quantified for each precordial lead (V1-V6) using custom software developed in MATLAB (Version 2012b, Mathworks, USA) as previously described [12,13]. The precordial leads were chosen for analysis because each is an independent unipolar recording unlike the six limb leads which are derived from only two independent lead pairs (i.e. leads I and II). In brief, QRSp for each lead represented the total number of abnormal QRS deflections that deviated from a smoothed QRS template of that lead.

**Fig 1:**
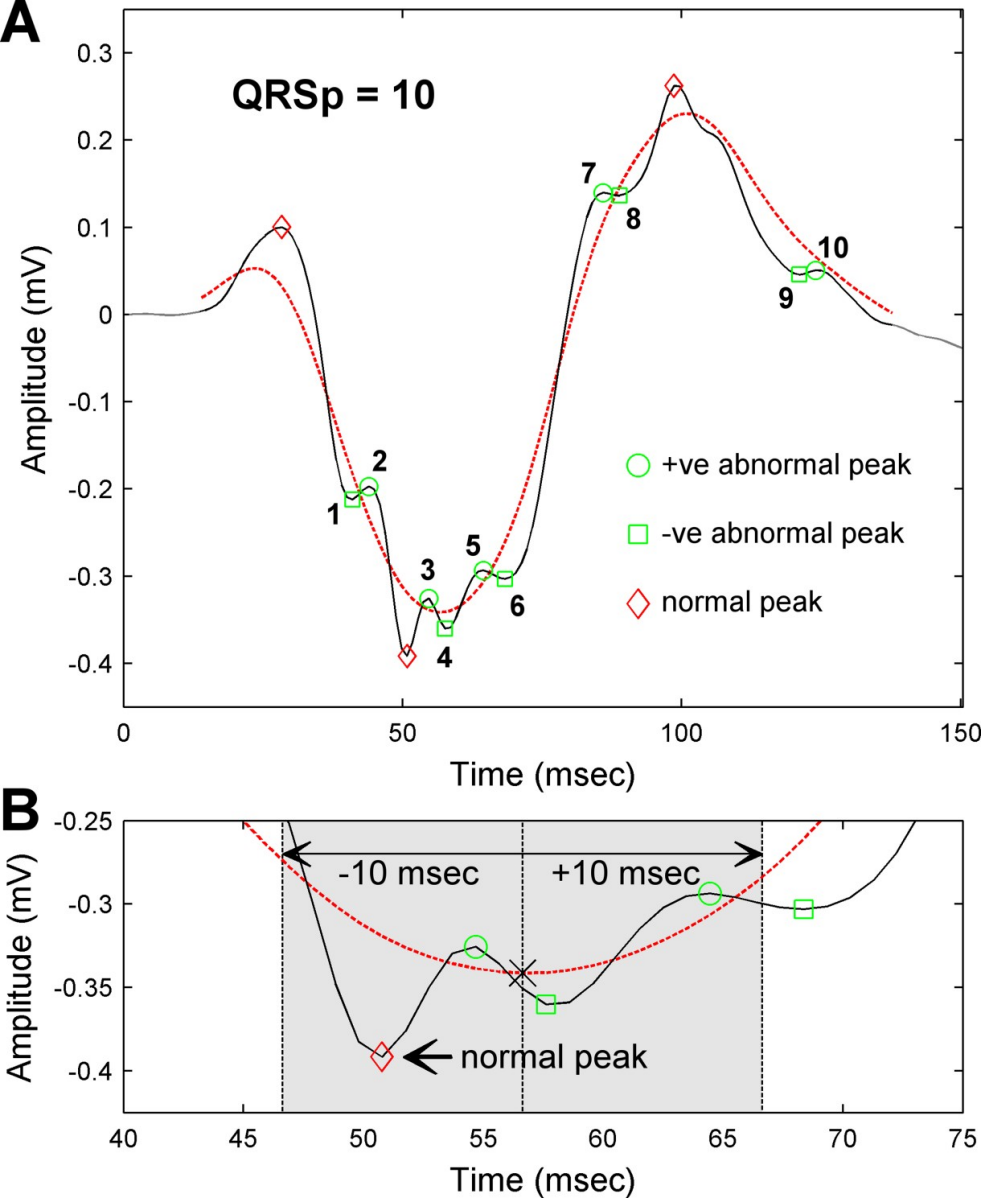
Quantification of QRSp. (A) Illustration of the QRSp method applied to lead V5 of a representative patient. Five positive (green circles) and five negative (green squares) abnormal QRS peaks are identified on the local QRS (lQRS; solid black line) after identifying three normal peaks (red diamonds) using the smoothed global QRS (gQRS; dashed red line). The number of positive and negative abnormal peaks is summed to produce a QRSp score of 10. (B) Illustrative example of normal QRS peak classification using a magnified view of the QRS complex in Panel A. A negative peak is identified on the smoothed gQRS (black x). The most negative peak on the lQRS within ±10ms of the gQRS peak (shaded area) is classified as normal.

Prior to QRSp detection, each lead was pre-processed to reduce noise and exclude irregular beats (e.g. noisy, paced, fused or ectopic beats) as described in the S1 Methods. The first 100 consecutive, non-excluded QRS complexes in the 3-minute recording were used to quantify QRSp. For each lead, QRSp was assessed in consecutive 10-beat windows incremented by a single beat from the first to the last of the 100 QRS complexes. We have previously shown that a 10-beat window provides sufficient signal averaging to reduce noise, while a longer 100-beat window actually reduces QRS peak detection [12]. This may arise from cyclic respiratory chest wall excursions, which can produce fluctuations in the amplitude of the precordial lead QRS complex. In each 10-beat window, abnormal QRS peaks were distinguished from normal QRS peaks by comparing two different filtered versions of the QRS complex (S1 Methods): (1) a smoothed moving average filtered 100-beat global QRS average (gQRS) and (2) a non-smoothed 10-beat local QRS average (lQRS). The smoothed gQRS complex contains the major components of the depolarizing wavefront and its local maxima and minima are considered to be normal QRS peaks. However, the non-smoothed lQRS retains minor perturbations that may relate to more localized conduction abnormalities in addition to the major components of the depolarizing wavefront. Thus, peaks detected on the lQRS that are not present on the gQRS are considered to be abnormal as illustrated in Fig 1. The QRSp for a 10-beat window was calculated as the total number of abnormal peaks identified in that window.

QRSp for each precordial lead (V1p -V6p) was computed as the greatest QRSp value found in at least 5 of the 90 individual 10-beat windows, an empirically derived threshold used to reduce the chances of a spurious result. QRSp Max and QRSp Mean were calculated for each patient as the maximum and mean of their six precordial lead QRSp values, respectively. Noise was assessed for each lead by computing the average ST segment root mean square noise value (RMS-ST) of all 100 beats [12].

### Functional endpoints

Response to CRT was evaluated functionally based on echocardiographic-derived LVEF in all patients. Two-dimensional echocardiography was performed according to standard clinical procedures within 3 months prior to CRT and 6–18 months post-CRT. LVEF was calculated from the apical 2- and 4-chamber views using the biplane Simpson’s method. Final interpretation was conducted by an echocardiographer blinded to the patient’s clinical and ECG characteristics. CRT response was defined as an absolute increase in LVEF of >5% between pre- and post-CRT assessments [15].

### Statistical analyses

Continuous variables are presented as mean ± standard deviation or median and interquartile range (25^th^-75^th^ percentiles) where appropriate. Student's *t* test or the Mann-Whitney U-test was used for unpaired comparison between CRT responders and non-responders. Categorical variables are presented as frequency or percentage and were compared by χ2 or Fisher's exact test. Amongst responder and non-responder groups, the paired t-test was used to compare continuous variables and McNemar’s test was used to compare categorical variables between baseline and follow-up. One-way analysis of variance was used to compare variables between the patients divided into 4 groups based on combined optimal QRSp and QRSd cut-points. Correlations were assessed using Pearson’s correlation coefficient.

Univariable and multivariable logistic regression analysis was used to assess the predictive value of clinical variables and QRSp Max for CRT response. Amongst the potential QRSp metrics, QRSp Max was chosen *a priori* as it was significantly different between responders and non-responders and provided an aggregate evaluation of abnormal QRS peaks across all the precordial leads. The multivariable model included predictors with a univariable significance level of P<0.1 as well as QRSd due to its known associations with CRT response. Regression results are presented as the odds ratio and 95% confidence interval (OR [95% CI]).

Receiver operating characteristic (ROC) curves were constructed for QRSp and QRSd as predictors of CRT response. For QRSp, the optimal cut-point was assessed using Youden’s index and the point closest to (0,1), while for QRSd, the conventionally accepted cut-point of 150ms was used [3]. The area under the paired ROC curves was compared between QRSp and QRSd using the DeLong test. Sensitivity and specificity were compared between QRSp and QRSd cut-points using NcNemar’s test, while positive and negative predictive value were compared using a weighted generalized score statistic.

All statistical analyses were performed using MATLAB (version 8.0, MathWorks, USA) or SPSS (version 20.0, SPSS Inc., USA). A two-sided P<0.05 was considered statistically significant, except when multiple comparisons were made amongst the family of QRSp variables (V1p, V2p, V3p, V4p, V5p, V6p, QRSp Max and QRSp Mean), in which cases a Bonferroni-corrected significance level of P<0.00625 was used to control for potential experiment-wise error.

## Results

### Patient characteristics and CRT response

Patient baseline characteristics are presented in Table 1. All patients had both pre- and post-CRT echocardiograms performed, with LV functional response to CRT observed in 28 (60%) patients. Median follow-up time for the post-CRT echocardiogram was 11.3 (7.4–14.7) months and was similar between responders and non-responders (11.7 (7.1–15.4) vs. 11.0 (7.5–13.8) months, p = 0.60). The percentage of biventricular pacing at follow-up was 96±6% and did not differ between response groups (96±6 vs. 97±7%, p = 0.61). The LVEF increased in responders (23.3±6.7 to 38.0±9.7%, p<0.001) but did not change in non-responders (23.4±8.2 to 24.2±7.9%, p = 0.42). While fewer responders had a history of AF than non-responders (7 vs. 32%, p = 0.047), all other clinical characteristics were similar between the groups. There was also no difference in CRT parameters between responders and non-responders (S1 Table).

**Table 1:** Patient baseline characteristics.

	Total Sample (N = 47)	CRT Non-Responder (N = 19)	CRT Responder (N = 28)	P
**Age, years**	62±14	58±16	64±11	0.11
**Male, n (%)**	30 (64)	12 (63)	18 (64)	1.00
**LVEF, %**	23±7	23±8	23±7	0.93
**Cardiomyopathy, n (%)**				1.00
**Ischemic**	16 (34)	6 (32)	10 (36)	
**Non-Ischemic**	31 (66)	13 (68)	18 (64)	
**NYHA Class, n (%)**				0.34
I*	1 (2)	1 (5)	0 (0)	
**II**	18 (38)	5 (26)	13 (46)	
**III**	25 (53)	12 (63)	13 (46)	
**IV**	3 (6)	1 (5)	2 (7)	
**History of AF**	8 (17)	6 (32)	2 (7)	**0.047**
**Creatinine (μmol/L)**	110±68	114±55	108±75	0.76
**eGFR (ml/min)**	66±22	62±23	69±22	0.30
**Medications**				
**β-blocker, n (%)**	46 (98)	19 (100)	27 (96)	1.00
**ACE Inhibitor/ARB, n (%)**	46 (98)	18 (95)	28 (100)	0.40
**Diuretic, n (%)**	42 (89)	18 (95)	24 (86)	0.64
**Digoxin, n (%)**	14 (30)	8 (42)	6 (21)	0.20
**Amiodarone, n (%)**	10 (21)	7 (37)	3 (11)	0.07
**Heart Rate, bpm**	69±17	69±19	70±15	0.88
**Native QRS Morphology, n (%)**				0.67
**LBBB**	41 (87)	16 (84)	25 (89)	
**RBBB/IVCD**	6 (13)	3 (16)	3 (11)	
**QRSd, ms**	173±32	164±30	179±32	0.11
**QRSd≥150ms, n (%)**	38 (81)	13 (68)	25 (89)	0.13
**fQRS, n (%)**	25 (53)	8 (42)	17 (61)	0.25
**QRSp**				
**V1p**	2.8±2.9	3.1±2.7	2.6±3.1	0.574
**V2p**	2.1±2.4	2.2±2.3	2±2.5	0.733
**V3p**	2.6±3.5	1.7±1.6	3.1±4.3	0.132
**V4p**	4±3.5	2.8±2.1	4.7±4	0.045
**V5p**	6.2±3.8	4.9±2.4	7±4.4	0.046
**V6p**	5.4±3.7	3.6±2.4	6.7±3.9	** 0.003†**
**QRSp Max**	7.8±3.4	5.9±2.2	9.1±3.5	** 0.001†**
**QRSp Mean**	3.6±2.1	2.9±1.2	4±2.5	0.085

ACE, angiotensin converting enzyme; AF, atrial fibrillation; ARB, angiotensin receptor blocker; CRT, cardiac resynchronization therapy; fQRS, fragmented QRS; IVCD, intraventricular conduction block; LBBB, left bundle branch block; LVEF, left ventricular ejection fraction; QRSd, QRS duration; QRSp, QRS peaks; QRSp Max, maximum of precordial lead QRSp values; QRSp Mean, mean of precordial lead QRSp values; RBBB, right bundle branch block; V1p-V6p, QRSp measured in leads V1-V6

*NYHA class I patient with LVEF<35%, QRSd>120ms and bradycardia indication for pacing

†QRSp variables below Bonferroni corrected significance level (p<0.00625).

### Baseline ECG characteristics and CRT response

**Fig 2:**
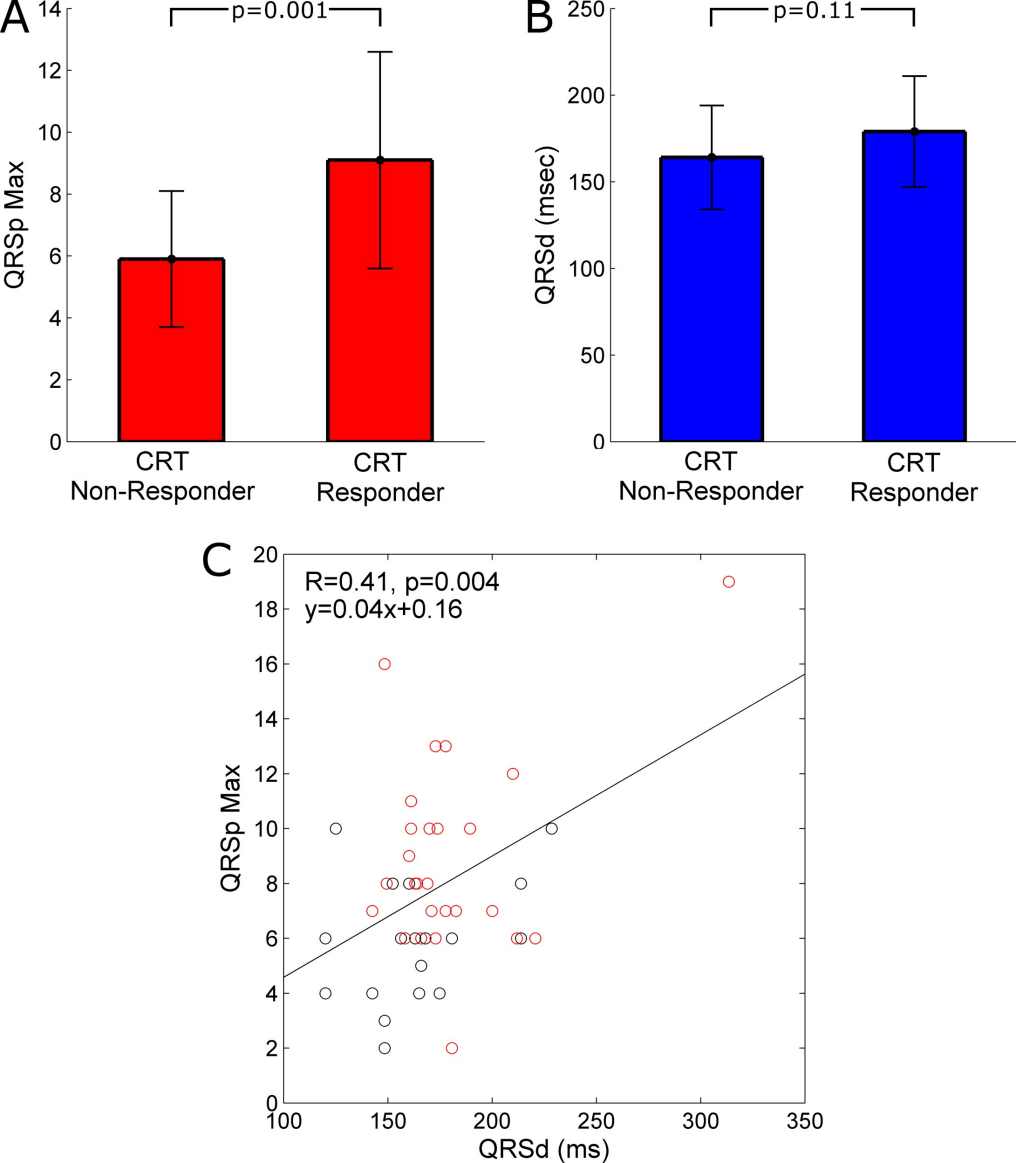
Comparison of QRSp and QRSd between CRT response groups (N = 47). Bar graphs comparing (A) QRSp Max and (B) QRSd between CRT responders and non-responders. (C) Scatter plot illustrating a significant (p<0.004) but weak (R = 0.41) linear correlation between the baseline QRSp Max and QRSd for all 47 CRT patients. The 28 CRT responders are highlighted with red circles.

The baseline ECG study was conducted at a median 1.8 (1.3–2.4) months post-CRT and occurred at similar times in responders and non-responders (1.8 (1.3–2.4) vs. 1.8 (1.3–2.3) months, p = 1.00). As presented in Table 1, baseline V6p (6.7±3.9 vs. 3.6±2.4, p = 0.003) and QRSp Max (Fig 2A; 9.1±3.5 vs. 5.9±2.2, p = 0.001) were greater in responders compared to non-responders. There was also a trend toward greater V4p, V5p and QRSp Mean in responders (p<0.1). However, there was no difference in baseline QRSd (Fig 2B; 179±32 vs. 164±30 ms, p = 0.11) or the proportion of patients with QRSd ≥150ms (89 vs. 68%, p = 0.13) between responders and non-responders. The prevalence of fQRS was similar between responders and non-responders (61 vs. 42%, p = 0.25). As illustrated in Fig 2C, baseline QRSp Max and QRSd were only weakly positively correlated (r = 0.41, p = 0.004). There was no difference in QRSp Max between patients with and without fQRS (8.4±3.6 vs. 7.1±3.1, p = 0.18), LBBB (7.6 vs. 9.2, p = 0.30) or AF (6.5 vs. 8.1, p = 0.23).

Importantly, RMS-ST noise did not relate to QRSp or CRT response. There was no correlation between the QRSp and average RMS-ST noise value of each precordial lead (V1: r = 0.10, p = 0.52; V2: r = -0.10, p = 0.52; V3: r = 0.00, p = 0.99; V4: r = -0.15, p = 0.33; V5: r = 0.00, p = 1.00; V6: r = 0.09, p = 0.55), suggesting that noise did not significantly contribute to the QRSp signal. There was also no difference in the precordial lead RMS-ST noise values between responders and non-responders (V1: 1.2±0.5 vs. 1.0±0.4μV, p = 0.13; V2: 1.1±0.4 vs. 0.9±0.3μV, p = 0.15; V3: 1.1±0.3 vs. 0.9±0.3μV, p = 0.09; V4: 1.1±0.3 vs. 1.0±0.3μV, p = 0.18; V5: 1.1±0.2 vs. 0.9±0.3μV, p = 0.13; V6: 1.0±0.2 vs. 0.9±0.3μV, p = 0.07).

Univariable and multivariable logistic regressions models for the prediction of CRT response are presented in Table 2. Univariable analysis revealed QRSp Max (OR [95% CI]: 1.58 [1.15–2.16], p = 0.005), QRSd ≥150ms (3.85 [0.83–17.9], p = 0.09), and history of AF (0.17 [0.03–0.94], p = 0.04) to be predictors of response to CRT. Multivariable analysis of the univariable predictors revealed QRSp Max to be the only independent predictor of CRT response in a model including QRSd as continuous variable (OR [95% CI]: 1.55 [1.11–2.16], p = 0.01; c-statistic = 0.84) and another including QRSd ≥150ms as a dichotomous variable (1.54 [1.11–2.14], p = 0.01; c-statistic = 0.82).

**Table 2:** Logistic regression analysis for prediction of CRT response (N = 47).

	Univariable Analysis		Multivariable Model 1*		Multivariable Model 2†	
	OR (95% CI)	*P*	OR (95% CI)	*P*	OR (95% CI)	*P*
**Follow-up Time, mos**	1.02 (0.95–1.10)	0.52	-	-	-	-
**Male**	1.05 (0.31–3.53)	0.94	-	-	-	-
**Non-Ischemic CM**	0.83 (0.24–2.87)	0.77	-	-	-	-
**History of AF**	**0.17 (0.03–0.94)**	**0.04**	0.15 (0.02–1.34)	0.09	0.18 (0.02–1.49)	0.11
**LVEF (per 5%)**	0.98 (0.66–1.48)	0.94	-	-	-	-
**LBBB**	1.56 (0.28–8.72)	0.61	-	-	-	-
**QRSd ≥150ms**	**3.85 (0.83–17.9)**	**0.09**	-	-	3.15 (0.41–24.4)	0.27
**QRSd (per 10ms)**	1.23 (0.95–1.58)	0.12	1.20 (0.87–1.65)	0.27	-	-
**fQRS**	2.12 (0.65–6.95)	0.21	-	-	-	-
**QRSp Max (per unit)**	**1.58 (1.15–2.16)**	**0.005**	**1.55 (1.11–2.16)**	**0.01**	**1.54 (1.11–2.14)**	**0.01**

AF, atrial fibrillation; CI, confidence interval; CM, cardiomyopathy; fQRS, fragmented QRS; LBBB, left bundle branch block; LVEF, left ventricular ejection fraction; OR, odds ratio; QRSd, QRS duration; QRSp Max, maximum of precordial lead QRS peaks values

*C-Statistic = 0.84

†C-Statistic = 0.82

### ROC analysis of QRSp and QRSd

**Fig 3:**
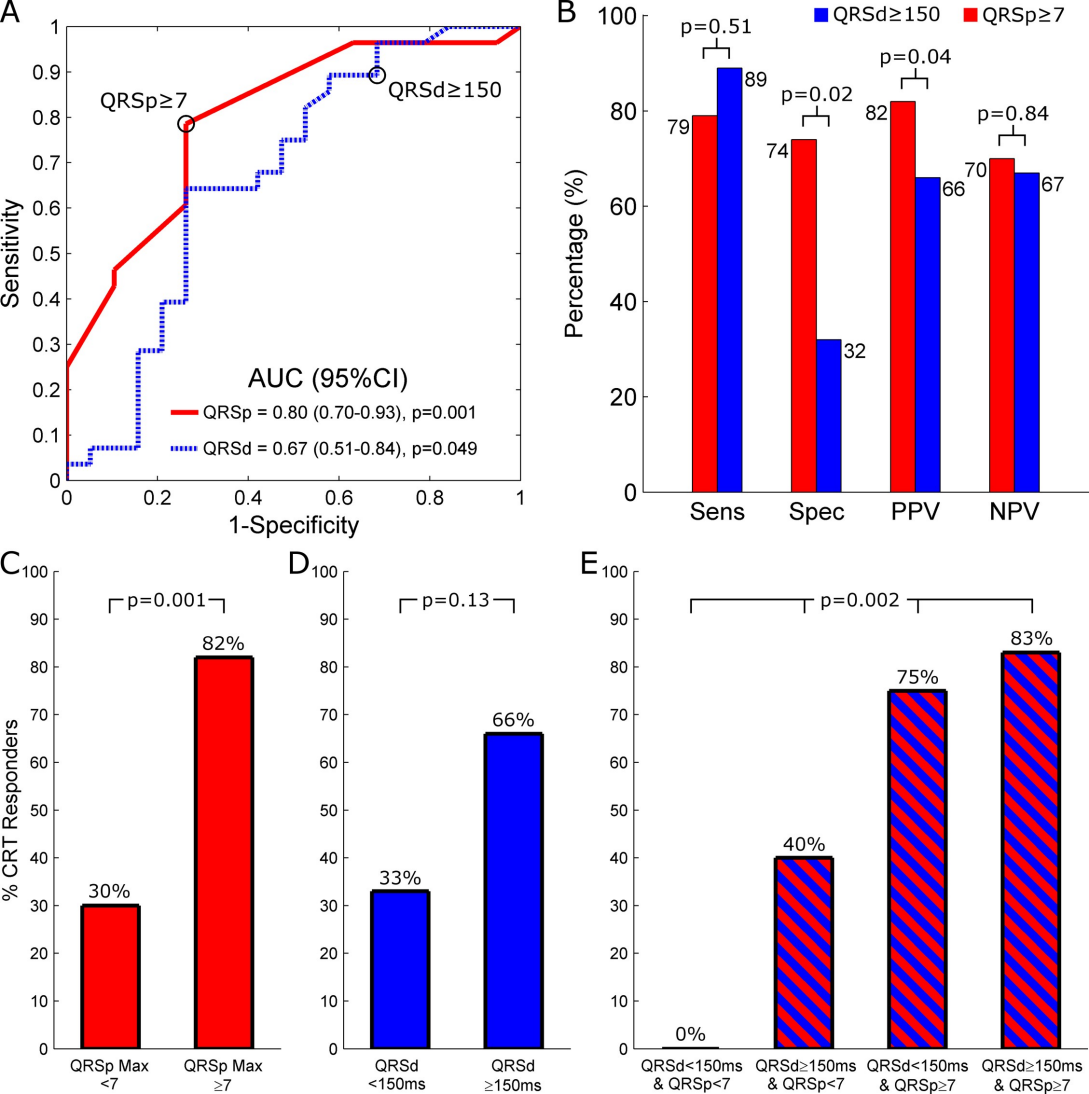
Performance of QRSp and QRSd in predicting CRT response (N = 47). (A) ROC curves for QRSp Max (red solid line) and QRSd (blue dashed line) as a predictor of CRT response. Black circles highlight the sensitivity and 1-specificity obtained by using a QRSp Max cutpoint ≥7 and a QRSd cutpoint ≥150ms. (B) Bar graphs comparing the prognostic performance of the QRSp Max and QRSd cut-points for identifying CRT responders. (C-E) Bar graphs comparing CRT responder rates between patients stratified by (C) the optimal QRSp Max cutpoint of ≥7, (D) the conventional QRSd cutpoint of ≥150ms, and (E) the combination of both these cutpoints. While nearly all patients with both QRS ≥150ms and QRSp ≥7 responded to CRT, there were no responders amongst patients with both a QRS <150ms and QRSp <7. AUC = area under the curve. 95% CI = 95% confidence interval. PPV = positive predictive value. NPV = negative predictive value.

Since intrinsic QRSd is an established marker of CRT response, ROC analysis was used to evaluate the performance characteristics of baseline QRSp Max compared to baseline QRSd (Fig 3A & 3B). Although the area under the ROC curve for identifying CRT responders was greater for QRSp Max (area under curve [95% CI]: 0.80 [0.70–0.93], p = 0.001) than for QRSd (0.67 [0.51–0.84], p = 0.049), the difference between them was not statistically significant (p = 0.21). For comparison with the conventional QRSd cutpoint of ≥150ms used to improve CRT selection, a QRSp Max cutpoint of ≥7 was selected as it was both the point closest to (0,1) on the ROC curve and the optimal cut-point defined by Youden’s index for identifying CRT responders. Using these cut-points, QRSp Max ≥7 achieved similar sensitivity (79 vs. 89%, p = 0.51) and negative predictive value (70 vs. 67%, p = 0.84), but greater specificity (74 vs. 32%, p = 0.02) and positive predictive value (82 vs. 66%, p = 0.04) than QRSd ≥150ms. The probability of CRT response was greater in patients with QRSp Max ≥7 than in those with <7 (82% [22 of 27] vs. 30% [6 of 20], p = 0.001; Fig 3C), but similar in patients with QRSd ≥150ms compared with those with <150ms (66% [25 of 38] vs. 33% [3 of 9], p = 0.13; Fig 3D). There was no difference in clinical characteristics between patients with a QRSp Max ≥7 and <7 (S2 Table).

### QRSp in patients with QRSd <150ms and ≥150ms

As illustrated in Fig 3E, the probability of CRT response was greatest in patients with QRSd ≥150ms and QRSp Max ≥7 (83% [19 of 23]), followed by QRSd <150ms and QRSp Max ≥7 (75% [3 of 4]), QRSd ≥150ms and QRSp Max <7 (40% [6 of 15]) and QRSd <150ms and QRSp Max <7 (0% [0 of 5]) (p = 0.002). These four categories also tracked structural remodeling, as the absolute LVEF improvement was most pronounced in patients with QRSd ≥150ms and QRSp Max ≥7 (Δ12.2±8.9%), followed by QRSd <150ms and QRSp Max ≥7 (Δ9.6±8.8%), QRSd ≥150ms and QRSp Max <7 (Δ7.3±8.8%) and QRSd <150ms and QRSp Max <7 (Δ-1.0±4.7) (p = 0.02). S3 Table summarizes additional patient and ECG characteristics of these 4 categories.

### ECG characteristics in follow-up and CRT response

**Fig 4:**
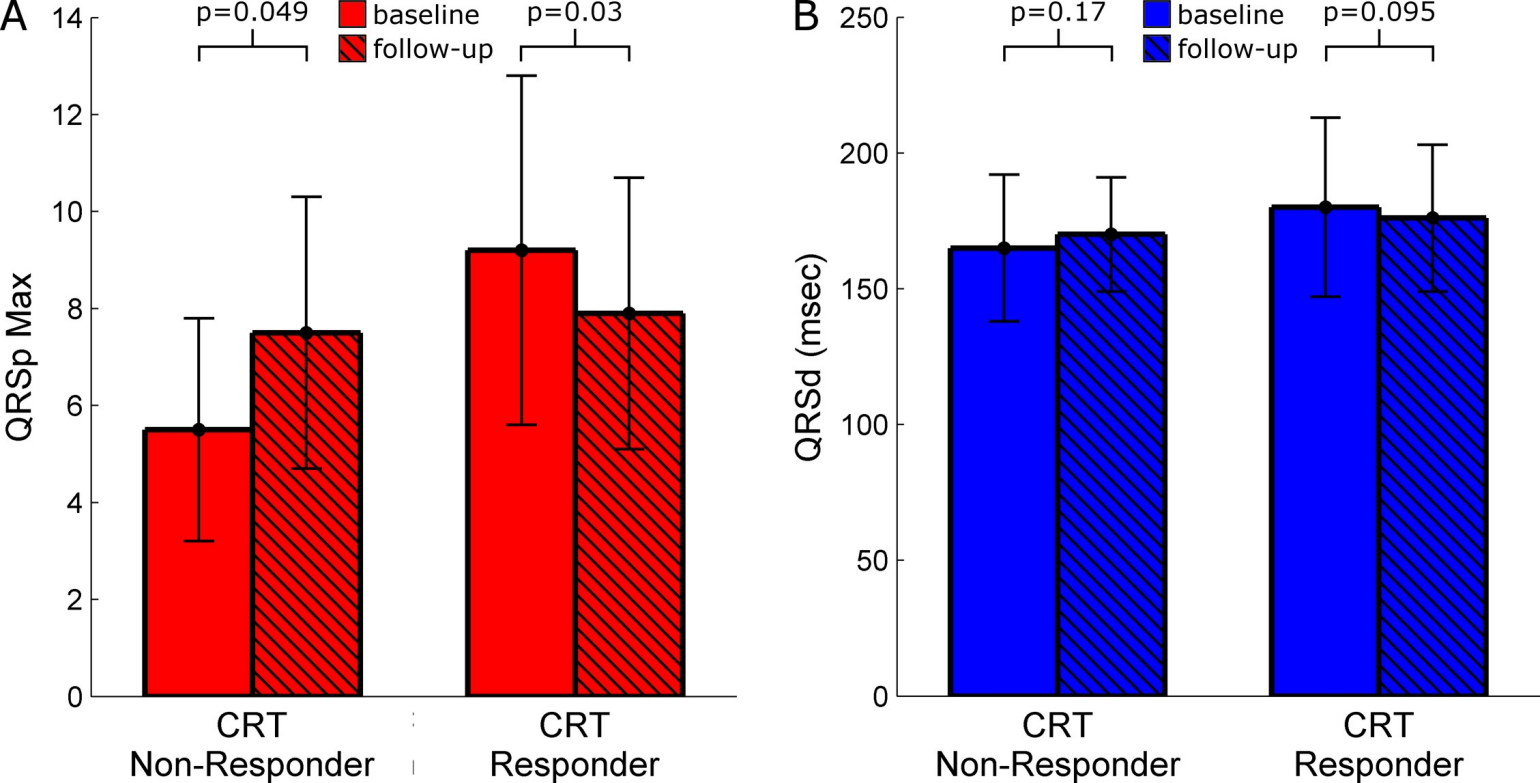
Comparison of QRSp and QRSd between baseline and follow-up in CRT responders and non-responders (N = 38). Bar graphs comparing changes between baseline and follow-up (A) QRSp Max and (B) QRSd in CRT responders and non-responders.

**Fig 5:**
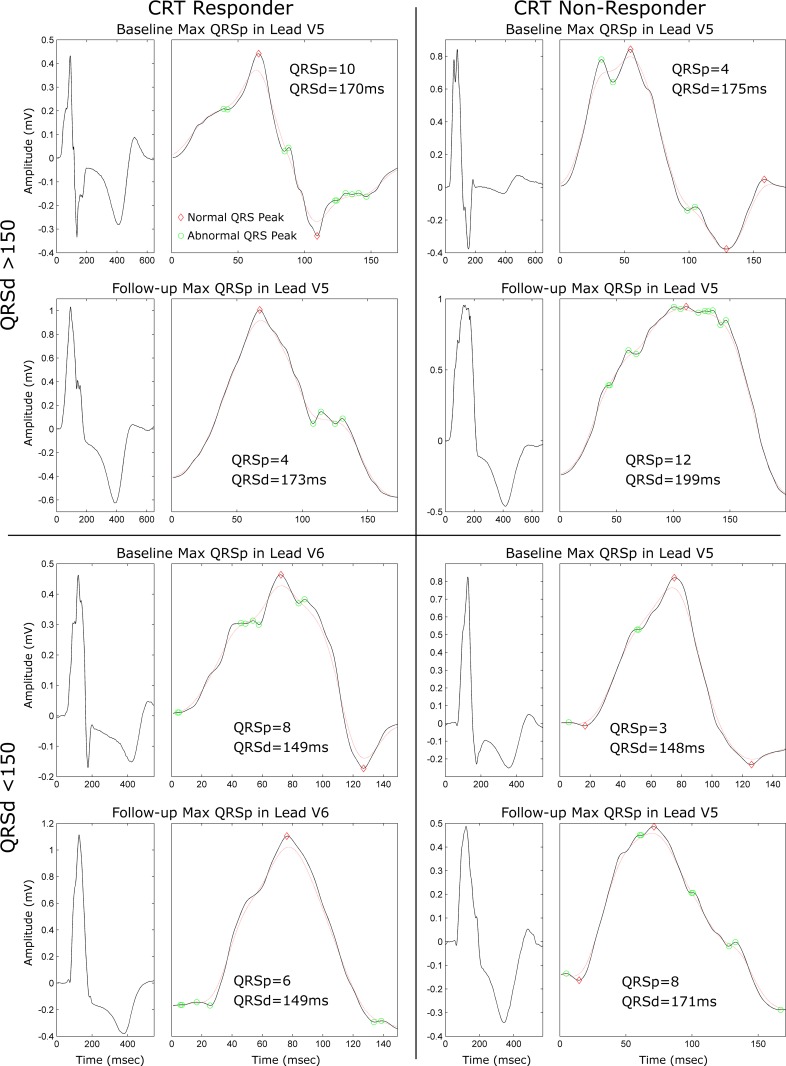
Examples of QRSp at baseline and follow-up in CRT responders and non-responders with baseline QRSd above and below 150ms. QRSp Max results at baseline and follow-up for a single 10-beat window from (top left) a CRT responder with a QRSd ≥150ms, (top right) a CRT non-responder with a QRSd ≥150ms, (bottom left) a CRT responder with a QRSd <150ms, and (bottom right) a CRT non-responder with a QRSd <150ms. Irrespective of their baseline QRSd, the CRT responders have greater baseline QRSp Max values than the non-responders. At post-CRT followup, the QRS complexes of the responders become smoother and their QRSp decreases, while the QRS complexes of non-responders become more fractionated and their QRSp increase. Solid black lines demarcate the lQRS and dashed red lines demarcate the gQRS. Normal peaks are annotated with red diamonds, and abnormal peaks are annotated with green circles.

Amongst the 38 patients with repeat ECG studies, the follow-up ECG was performed at a median 8.3 (7.4–11.9) months post-CRT with a trend toward being earlier in responders than non-responders (7.9 (6.7–10.6) vs. 11.6 (7.8–14.6), p = 0.06). Fig 4 illustrates changes in QRSp Max and QRSd from baseline to follow-up in CRT responders and non-responders. QRSp Max decreased in responders (9.2±3.6 vs. 7.9±2.8, p = 0.03) but increased in non-responders (5.5±2.3 vs. 7.5±2.8, p = 0.049). While there was a trend toward reduction of QRSd in responders (180±33 vs. 176±27, p = 0.095), QRSd in non-responders remained similar (165±27 vs. 170±31, p = 0.17). There was no change in the prevalence of fQRS amongst responders (59 vs. 81%, p = 0.15) or non-responders (45 vs. 64%, p = 0.50). Fig 5 (left panel) illustrates two CRT responders with QRSd above and below 150ms that have a baseline QRSp Max ≥7, which decreases in follow-up; while the right panel shows two CRT non-responders with QRSd above and below 150ms that have a baseline QRSp Max <7, which increases in follow-up. From baseline to follow-up, it was generally observed that the QRS complex became visually smoother in responders but more fragmented in non-responders as is seen in these representative examples.

## Discussion

The main findings of our study are as follows: (i) QRSp independently predicts functional CRT response in cardiomyopathy patients, unlike QRSd, LBBB or fQRS, (ii) QRSp ≥7 identifies CRT responders with greater accuracy than QRSd ≥150ms or fQRS, and (iii) QRSp decreases in CRT responders while increasing in CRT non-responders, whereas QRSd and fQRS do not change irrespective of CRT response.

### Predicting CRT response with QRSp

Electrical conduction delay in the LV causes dyssynchronous activation, which may respond to CRT. Currently, electrical dyssynchrony assessment for CRT eligibility is based solely on the QRSd ≥120ms and LBBB morphology [1]. While these contemporary criteria have high sensitivity, they lack specificity to predict CRT response [4]. Moreover, in patients with QRSd <150ms, there are limited data on markers of electrical dyssynchrony. Recognition of patients prior to device implantation who are likely to achieve benefit or are at a heightened risk of non-response to CRT has been challenging.

Our study for the first time shows that QRSp independently predicts functional CRT response in cardiomyopathy patients after adjusting for QRSd, while LBBB and fQRS were not predictive. For each QRS peak detected, the odds of CRT response increase by 1.6-fold. QRSp ≥7 identified CRT responders with similar sensitivity as QRSd, but with significantly greater specificity (74 vs. 32%) and positive predictive value (82 vs. 66%). Importantly, QRSp discriminated CRT responders from non-responders among patients with QRSd >150ms as well as those with QRSd <150ms. Patients with QRSd ≥150ms who also had QRSp of ≥7 had a >2-fold higher probability of attaining functional CRT response (83% vs. 40%) and a larger LVEF improvement compared to those with QRSp <7. In addition, although a minority of our study patients had QRSd <150ms, QRSp <7 in this group predicted 100% non-response to CRT.

### Change in QRSd, QRSp, fQRS and LVEF with long-term CRT

Electrical resynchronization of the ventricles with CRT is mediated by fusion of RV and LV propagating wavefronts, which reduces global activation time and mitigates conduction block [5]. This may result in an acute narrowing in the paced QRSd and a reduction in paced fQRS prevalence compared to that in native QRS [4,6]. With long-term CRT, there is evidence of reverse electrical remodeling amongst CRT responders. Narrowing of the native QRSd has been reported in both functional and clinical CRT responders [16], while complete reversion of LBBB has been linked with super-response [17]. In a prospective study of 85 CRT patients with LBBB, Sebag et al [16] reported a decrease in intrinsic QRSd from 168±20ms pre-CRT to 149±31ms at 1-year post-CRT, which was associated with a greater rate of LVEF improvement. In addition, Celikyurt et al [18] observed a reduction in the prevalence of native fQRS amongst CRT patients with LBBB who demonstrated a decrease in LV end systolic volume >15% at 6 months post-CRT. Electrical remodeling in CRT responders may arise from structural and molecular changes in the cardiac electric substrate. Besides restoration of the cardiomyocyte size and reduction in collagen fraction, there is improvement in kinetics of intracellular calcium cycling with up-regulation of sarcoplasmic reticulum calcium ATPase 2α and ryanodine receptor, and sodium-calcium exchanger levels in those with LVEF improvement post CRT [19,20]. No significant changes in molecular profile are observed in non-responders. In line with these data, we found a significant reduction in QRSp in patients who had improvement in LVEF, but an increase in QRSp in non-responders. In contrast, changes in native QRSd were less evident in our study, although there was a trend toward a decrease in responders, but no change in non-responders. We also did not observe any change in fQRS prevalence in CRT responders or non-responders. Therefore, long-term reduction in QRSp alone with CRT pacing appears be an important prognostic marker for mechanical recovery after CRT, whereas an increase in QRSp may arise from adverse remodeling possibly induced by CRT.

### QRS fragmentation and ventricular remodeling

Fragmentation of the QRS complex on the standard 12-lead ECG reflects conduction delay and abnormal wavefront propagation caused by fibrotic infiltration and/or ventricular ion-current remodeling [21]. A linkage between fQRS and myocardial scarring was originally described in patients with narrow QRS (<120ms) but has since been extended to patients with wide QRS, paced QRS and premature ventricular beats [14]. In this regard, native fQRS has been associated with major adverse events in patients with both ischemic and non-ischemic heart disease, with increased risk of ventricular arrhythmias and death [7].

Amongst the CRT population, paced fQRS has also been associated with future ventricular tachyarrhythmia events and sudden cardiac death in patients with non-ischemic cardiomyopathy [6]. Paradoxically, in the same study and another that included both ischemic and non-ischemic cardiomyopathy, CRT patients with native fQRS had equivalent mortality risk compared to those without fQRS [6,10]. Native fQRS has also been associated with echocardiographic-derived inter- and intra-ventricular dyssynchrony in two observational studies of 286 cumulative patients with non-ischemic cardiomyopathy and QRSd<120ms [8,9]. However, in those with a wider QRSd, mechanical dyssynchrony appears to be equally prevalent in patients with and without native fQRS [11]. Only a few studies have evaluated the relation of native fQRS to functional CRT response and their results are conflicting. In a retrospective study of 232 CRT patients, Rickard et al [10] demonstrated fQRS in 21% of the cohort and there was no difference in LVEF improvement or LV end diastolic volume reduction between those with and without fQRS. In a smaller prospective study of 53 patients of whom 31% manifested fQRS, the absence of fQRS was an independent predictor of functional CRT response (OR 1.55, p = 0.028) [11].

In our study, QRSp independently predicted functional CRT response and a higher QRSp was associated with greater LVEF recovery in both patients with QRSd <150ms as well as those with QRSd ≥150ms. On the other hand, we did not find fQRS to be associated with functional CRT response. This discrepancy from the above-mentioned fQRS-based studies suggests that the quantitative evaluation of QRS fragmentation with QRSp provides a more specific measure of electrical dyssynchrony. QRSp is a quantitative metric measured from high-resolution ECG recordings (≥1000Hz). The abnormal QRS peaks counted are typically not visually apparent (amplitude ~6uV, width ~6ms) and do not include larger “normal” peaks that define the broad shape of the QRS complex [13]. In contrast, fQRS is a qualitative metric (i.e. present or absent) measured from standard low-resolution ECG recordings (<1000 Hz) that considers only larger visually discernible QRS deflections, which may include the normal peaks as defined by our algorithm. We observed no difference in QRSp between patients with and without fQRS, which further highlights the difference in myocardial substrate being evaluated by these two metrics.

### Limitations

Our sample size is limited and effectively that of a pilot study; however, significant differences in QRSp between responders and non-responders to CRT were still demonstrable. As the proportion of patients with QRSd <150ms was low, applicability of the QRSp metric for determining CRT-eligibility in these patients should be confirmed in a larger prospective study. Second, we could not characterize whether late reduction in QRSp was principally due to reverse cardiac remodeling through CRT or co-interventions such as medical therapy for heart failure, but medical therapy had been optimized prior to CRT implantation and was systematically adjusted post-implant in all patients as needed per heart failure practice guidelines [22]. Third, baseline QRSp was evaluated a median of 1.8 months post CRT, which coincided with the patient’s early postoperative follow-up, rather than pre CRT. However, this time interval is unlikely to be associated with CRT-induced structural remodeling as demonstrated by Cvijic et al [23] where LV end systolic volume did not change before 3 months of CRT and native QRSd decreased by a median of only 5ms after 1 month of CRT. Regardless, our functional CRT responder rate was similar to prior studies [16] and QRSp remodeling still tracked functional CRT response. Fourth, AF may be a potential confounder in our study as the proportion of patients with AF was greater in CRT non-responders than responders and AF was a predictor of functional CRT response in univariable modelling. However, AF was not a predictor of functional CRT response in multivariable modelling when QRSd ≥150ms and QRSp Max were also included. The utility of QRSp in predicting functional CRT response in patients with AF merits further study in a larger, prospective cohort. Finally, we did not evaluate the relationship between QRSp and myocardial scar burden, nor the effect of QRSp remodeling with CRT on reducing ventricular arrhythmia burden and mortality, but these also merit further study in a larger cohort.

### Conclusion

Automated quantification of abnormal QRS peaks from high-resolution ECGs is an independent predictor of CRT response in patients with cardiomyopathy. QRSp ≥7 identifies CRT responders with similar sensitivity as QRSd, but with significantly greater specificity. For each additional QRSp detected, the odds of CRT response increase by 1.6-fold. In addition, long-term change in this novel marker of electrical dyssynchrony tracks mechanical recovery in CRT responders. Larger CRT cohort studies are warranted to confirm these findings and the utility of QRSp in improving CRT selection and ventricular arrhythmia prognostication, particularly in those with QRSd <150ms and RBBB or intraventricular conduction delay.

## Supporting information

S1 Methods Supplementary methods. (DOCX)

S1 Table CRT parameters. (DOCX)

S2 Table Baseline clinical characteristics in patients with QRSp <7 and ≥7. (DOCX)

S3 Table LVEF, QRSd and QRSp characteristics in patients stratified by QRSd ≥150ms and QRSp ≥7. (DOCX)
