# Supplementary material for: Quantification of abnormal QRS peaks predicts response to cardiac resynchronization therapy and tracks structural remodeling
Source: PLoS One. 2019 Jun 6;14(6):e0217875. doi: 10.1371/journal.pone.0217875 (PMC6553860; doi:10.1371/journal.pone.0217875)
Supplement: S1 Methods — (DOCX) [file pone.0217875.s001.DOCX]

**Supplementary Methods**

**ECG Preprocessing**

Prior to QRSp quantification each lead was pre-processed as follows. First, a low noise-QRS template was manually defined by a trained observer blinded to CRT outcomes. Using the peak of the R-wave as a fiducial point, each QRS complex in the 3-minute recording was roughly aligned to the QRS template, and further refined by identifying the point of maximum cross-correlation with the QRS template in a window from 25 ms before to 25 ms after the initial alignment position. QRS templating and alignment were done separately for each lead. To eliminate non-sinus beats, complexes that were <90% similar to the morphology of the template complex were excluded [1]. Second, to eliminate noisy beats, QRS complexes with a ST segment root mean square noise value >10µV were excluded, which minimizes false QRS peak detection as previously shown [2]. Third, the ECG was denoised using a 150Hz bidirectional low pass filter (4^th^ order Butterworth) to attenuate high frequency noise [3], and then cubic spline corrected to remove baseline wander [4].

**gQRS and lQRS Construction and QRS Peak Classification**

For a 10 beat window, normal and abnormal QRS peaks were classified by comparing two different filtered versions of the QRS complex: (1) a smoothed global QRS average (gQRS) and (2) a non-smoothed local QRS average (lQRS). The gQRS was generated by applying a 15-point bidirectional moving average filter to the ECG and then signal averaging all 100 filtered QRS complexes. This produces a smoothed QRS complex with low frequency contours that portrays the major components of the depolarizing wavefront. Thus, all local maxima and minima in the gQRS are considered to be normal QRS peaks. In contrast, the lQRS was generated by signal averaging 10 consecutive QRS complexes within the 10-beat window without applying the additional moving average filter that was used to construct the gQRS. Since the lQRS is not smoothed like the gQRS, it not only contains the major components of the depolarizing wavefront but also retains minor perturbations that may relate to more localized conduction abnormalities. Accordingly, the local maxima and minima in the lQRS include both normal and abnormal QRS peaks. As illustrated in Figure 1, for a 10-beat window, the peaks on the lQRS corresponding, within 10ms, to those on the gQRS were classified as normal, while those not corresponding to gQRS peaks were considered abnormal.

**References**

1. Bobbs SE, Schmitt NM, Ozemek HS. QRS detection by template matching using real-time correlation on a microcomputer. *Journal of clinical engineering*. 1984; 9:197-212.

2. Suszko AM, Dalvi R, Das M, Chauhan VS. Quantifying abnormal QRS peaks using a novel time-domain peak detection algorithm: Application in patients with cardiomyopathy at risk of sudden death. *2015 IEEE International Conference on Electro/Information Technology (EIT)*. 2015; 020-024.

3. Morita H, Kusano KF, Miura D, Nagase S, Nakamura K, Morita ST, et al. Fragmented QRS as a marker of conduction abnormality and a predictor of prognosis of Brugada syndrome. *Circulation*. 2008; 118:1697-704.

4. Meyer CR, Keiser HN. Electrocardiogram baseline noise estimation and removal using cubic splines and state-space computation techniques. *Comput Biomed Res*. 1977; 10:459-70.
