# Supplementary material for: Quantification of abnormal QRS peaks predicts response to cardiac resynchronization therapy and tracks structural remodeling
Source: PLoS One. 2019 Jun 6;14(6):e0217875. doi: 10.1371/journal.pone.0217875 (PMC6553860; doi:10.1371/journal.pone.0217875)
Supplement: S1 Table — (DOCX) [file pone.0217875.s002.DOCX]

**S1 Table. CRT parameters.**

|  | **Total Sample (N=47)** | **CRT Non-Responder (N=19)** | **CRT Responder (N=28)** | **P** |
| --- | --- | --- | --- | --- |
| **LV Circumferential Lead Position, n (%)** |  |  |  | 0.17 |
| **Lateral** | 10 (21) | 6 (32) | 4 (14) |  |
| **Posterolateral** | 32 (68) | 10 (53) | 22 (79) |  |
| **Septal** | 5 (11) | 3 (16) | 2 (7) |  |
| **LV Apico-Basal Lead Position, n (%)** |  |  |  | 0.29 |
| **Basal** | 14 (30) | 7 (37) | 7 (25) |  |
| **Mid** | 32 (68) | 11 (58) | 21 (75) |  |
| **Apical** | 1 (2) | 1 (5) | 0 (0) |  |
| **LV Pacing Configuration, n (%)** |  |  |  | 0.77 |
| **Bipolar** | 25 (53) | 11 (58) | 14 (50) |  |
| **Extended Bipolar** | 22 (47) | 8 (42) | 14 (50) |  |
| **Sensed AV Delay, ms** | 112±18 | 111±14 | 113±20 | 0.78 |
| **Paced AV Delay, ms** | 152±24 | 158±19 | 148±25 | 0.22 |
| **LV to RV Delay** | 35±14 | 32±13 | 36±15 | 0.38 |
| **BiV Pacing, %** | 96±6 | 96±6 | 97±7 | 0.61 |

AV, atrioventricular; BiV, biventricular; CRT, cardiac resynchronization therapy; LV, left ventricular; RV, right ventricular
