# Supplementary material for: Quantification of abnormal QRS peaks predicts response to cardiac resynchronization therapy and tracks structural remodeling
Source: PLoS One. 2019 Jun 6;14(6):e0217875. doi: 10.1371/journal.pone.0217875 (PMC6553860; doi:10.1371/journal.pone.0217875)
Supplement: S2 Table — (DOCX) [file pone.0217875.s003.DOCX]

**S2 Table. Baseline clinical characteristics in patients with QRSp <7 and ≥7.**

|  | **Total Sample (N=47)** | **QRSp <7 (N=20)** | **QRSp ≥7 (N=27)** | **P** |
| --- | --- | --- | --- | --- |
| **Age, years** | 62±14 | 60±17 | 63±11 | 0.56 |
| **Male, n (%)** | 30 (64) | 12 (60) | 18 (67) | 0.76 |
| **LVEF, %** | 23±7 | 23±8 | 24±7 | 0.64 |
| **Cardiomyopathy, n (%)** |  |  |  | 0.12 |
| **Ischemic** | 16 (34) | 4 (20) | 12 (44) |  |
| **Non-Ischemic** | 31 (66) | 16 (80) | 15 (56) |  |
| **NYHA Class, n (%)** |  |  |  | 0.67 |
| **I†** | 1 (2) | 1 (5) | 0 (0) |  |
| **II** | 18 (38) | 8 (40) | 10 (37) |  |
| **III** | 25 (53) | 10 (50) | 15 (56) |  |
| **IV** | 3 (6) | 1 (5) | 2 (7) |  |
| **History of AF** | 8 (17) | 4 (20) | 4 (15) | 0.71 |
| **Creatinine (µmol/L)** | 110±68 | 118±92 | 105±43 | 0.56 |
| **eGFR (ml/min)** | 66±22 | 67±23 | 66±22 | 0.82 |
| **Medications** |  |  |  |  |
| **β-blocker, n (%)** | 46 (98) | 20 (100) | 26 (96) | 1.00 |
| **ACE Inhibitor/ARB, n (%)** | 46 (98) | 20 (100) | 26 (96) | 1.00 |
| **Diuretic, n (%)** | 42 (89) | 17 (85) | 25 (93) | 0.64 |
| **Digoxin, n (%)** | 14 (30) | 6 (30) | 8 (30) | 1.00 |
| **Amiodarone, n (%)** | 10 (21) | 5 (25) | 5 (19) | 0.72 |
| **Heart Rate, bpm** | 69±17 | 72±19 | 67±15 | 0.39 |
| **Native QRS Morphology, n (%)** |  |  |  | 0.22 |
| **LBBB** | 41 (87) | 19 (95) | 22 (82) |  |
| **RBBB/IVCD** | 6 (13) | 1 (5) | 5 (19) |  |
| **QRSd, ms** | 173±32 | 167±27 | 177±35 | 0.28 |
| **QRSd≥150ms, n (%)** | 38 (81) | 15 (75) | 23 (85) | 0.47 |
| **fQRS, n (%)** | 25 (53) | 9 (45) | 16 (59) | 0.39 |

ACE, angiotensin converting enzyme; AF, atrial fibrillation; ARB, angiotensin receptor blocker; CRT, cardiac resynchronization therapy; fQRS, fragmented QRS; IVCD, intraventricular conduction block; LBBB, left bundle branch block; LVEF, left ventricular ejection fraction; QRSd, QRS duration; QRSp, QRS peaks
