# Supplementary material for: Quantification of abnormal QRS peaks predicts response to cardiac resynchronization therapy and tracks structural remodeling
Source: PLoS One. 2019 Jun 6;14(6):e0217875. doi: 10.1371/journal.pone.0217875 (PMC6553860; doi:10.1371/journal.pone.0217875)
Supplement: S3 Table — (DOCX) [file pone.0217875.s004.DOCX]

**S3 Table. LVEF, QRSd and QRSp characteristics in patients stratified by QRSd ≥150ms and QRSp ≥7.**

|  | **QRSd <150ms**  **& QRSp <7**  **(N=5)** | **QRSd ≥150ms**  **& QRSp <7**  **(N=15)** | **QRSd <150ms**  **& QRSp ≥7**  **(N=4)** | **QRSd ≥150ms**  **& QRSp ≥7**  **(N=23)** | **P** |
| --- | --- | --- | --- | --- | --- |
| **Responders, n (%)** | 0 (0) | 6 (40) | 3 (75) | 19 (83) | 0.002 |
| **Baseline LVEF, %** | 30±9 | 20±5 | 28±6 | 23±7 | 0.02 |
| **Follow-up LVEF, %** | 29±11 | 28±10 | 38±9 | 35±12 | 0.14 |
| **Δ LVEF, %** | -1.0±4.7 | 7.3±8.8 | 9.6±8.8 | 12.1±8.9 | 0.02 |
| **Baseline QRSd, ms** | 136±15 | 178±21 | 141±11 | 184±34 | 0.002 |
| **Baseline QRSp** |  |  |  |  |  |
| **V1p** | 1.6±1.7 | 2.1±2.0 | 4.5±4.2 | 3.3±3.3 | 0.31 |
| **V2p** | 1.2±1.1 | 1.3±1.5 | 1.3±1.9 | 2.9±2.9 | 0.16 |
| **V3p** | 1.2±1.8 | 2.0±2.0 | 0.5±1.0 | 3.6±4.5 | 0.22 |
| **V4p** | 1.6±1.7 | 2.7±1.8 | 3.0±1.2 | 5.4±4.2 | 0.03 |
| **V5p** | 2.2±1.5 | 4.6±1.7 | 9.5±5.0 | 7.5±4.0 | 0.002 |
| **V6p** | 2.6±1.9 | 3.7±2.0 | 5.5±2.4 | 7.2±4.2 | 0.007 |
| **QRSp Max** | 3.8±1.5 | 5.4±1.2 | 10.3±4.0 | 9.8±2.8 | <0.001 |
| **QRSp Mean** | 1.7±0.5 | 2.6±0.9 | 3.8±0.5 | 4.6±2.5 | 0.005 |

LVEF, left ventricular ejection fraction; QRSd, QRS duration; QRSp, QRS peaks; QRSp Max, maximum of precordial lead QRSp values; QRSp Mean, mean of precordial lead QRSp values; RBBB, right bundle branch block; V1p-V6p, QRSp measured in leads V1 through V6
